# Validation of the MEDSAIL Tool to Screen for Capacity to Live Safely and Independently in Nursing Home Residents

**DOI:** 10.1093/geroni/igaa057.145

**Published:** 2020-12-16

**Authors:** Whitney Mills, Mark Kunik, P Adam Kelly, Nancy Wilson, Steven Starks, Ali Asghar-Ali, Aanand Naik

**Affiliations:** 1 Providence VA Medical Center, Providence, Rhode Island, United States; 2 Center for Innovations in Quality, Effectiveness, and Safety (iQuest), Houston, Texas, United States; 3 Tulane University, New Orleans, Louisiana, United States; 4 Baylor College of Medicine, Houston, Texas, United States; 5 University of Houston, Houston, Texas, United States

## Abstract

Capacity for safe and independent living (SAIL) refers to an individual’s ability to solve problems associated with everyday life and perform activities necessary to live independently. Little guidance exists on the assessment of capacity for SAIL among nursing home residents. As a result, capacity for SAIL is not fully considered in the development of discharge plans to ensure safety and independence in the community. The Making and Executing Decisions for Safe and Independent Living (MEDSAIL) tool was developed to screen for capacity for SAIL among community-dwelling older adults. In this cross sectional pilot study, we tested the validity of MEDSAIL for use with nursing home residents. Participants were twenty-four residents of a Veterans Health Affairs nursing home. Exclusion criteria were cognitive impairment too severe to complete the protocol, diagnosis of serious mental illness or developmental disability, inability to hear, and inability to communicate verbally. Participants completed two assessments: the MEDSAIL interview administered by a research assistant and the criterion standard capacity interview administered by a geriatric psychiatrist. We examined internal consistency, convergent validity, divergent validity, and criterion-based validity. Five of seven MEDSAIL scenarios approximated acceptable levels of internal consistency (α>0.70). MEDSAIL scores were positively correlated with the criterion standard (0.88, p=0.001), and the Wilcoxon Rank Sum Test statistic was also statistically significant (p=0.001). MEDSAIL has promise as a user-friendly brief screening tool in nursing homes to understand resident capacity for SAIL and to inform development of discharge plans to keep the resident safe and independent in the community.

